# Recurrent Spiking Networks Solve Planning Tasks

**DOI:** 10.1038/srep21142

**Published:** 2016-02-18

**Authors:** Elmar Rueckert, David Kappel, Daniel Tanneberg, Dejan Pecevski, Jan Peters

**Affiliations:** 1 Intelligent Autonomous Systems Lab, Technische Universität Darmstadt, 64289, Germany; 2 Institute for Theoretical Computer Science, Technische Universität Graz, 8020, Austria; 3 Robot Learning Group, Max-Planck Institute for Intelligent Systems, Tuebingen, 72076, Germany

## Abstract

A recurrent spiking neural network is proposed that implements planning as probabilistic inference for finite and infinite horizon tasks. The architecture splits this problem into two parts: The stochastic transient firing of the network embodies the dynamics of the planning task. With appropriate injected input this dynamics is shaped to generate high-reward state trajectories. A general class of reward-modulated plasticity rules for these afferent synapses is presented. The updates optimize the likelihood of getting a reward through a variant of an Expectation Maximization algorithm and learning is guaranteed to convergence to a local maximum. We find that the network dynamics are qualitatively similar to transient firing patterns during planning and foraging in the hippocampus of awake behaving rats. The model extends classical attractor models and provides a testable prediction on identifying modulating contextual information. In a real robot arm reaching and obstacle avoidance task the ability to represent multiple task solutions is investigated. The neural planning method with its local update rules provides the basis for future neuromorphic hardware implementations with promising potentials like large data processing abilities and early initiation of strategies to avoid dangerous situations in robot co-worker scenarios.

Probabilistic inference has emerged as a promising framework for solving Markov decision problems (*probabilistic planning*)^1^,^2^. In parallel to this development, the probabilistic inference perspective has also been successfully used in cognitive science and neuroscience for modeling how biological organisms solve planning problems^3^,^4^,^5^,^6^. However, it was not clear how probabilistic planning can be implemented in neural substrates with biologically realistic learning rules. Here, we show that probabilistic planning can be implemented and learned by recurrent networks of spiking neurons such that the generated spike sequences realize mental plans.

Recently, it was shown that recurrent spiking networks can implement *Bayesian filtering* and are able to learn a generative model of temporal sequences from presented examples drawn from a target distribution (i.e., the distribution that has generated the samples)^7^,^8^,^9^. Training was realized through synaptic plasticity rules without supervision. After learning, when running freely, the neural networks effectively perform forward sampling in the dynamic Bayesian network representing the target distribution. For solving a *planning* problem, however, it is necessary to sample from the posterior distribution conditioned on receiving a reward. In other words, forward sampling needs to integrate future rewards propagating backward in time in the dynamic Bayesian network.

We consider a recurrent network of stochastic spiking neurons, which without any input, samples sequences through forward sampling from the corresponding dynamic Bayesian network. We show that by injecting appropriate input in the network from a layer of task-related *context neurons*, it can generate samples from the posterior distribution conditioned on getting a reward. Optimal reward-modulated Hebbian learning rules are derived that implement planning as probabilistic inference in the spiking network through iterative local updates. The recurrent dynamics of the neural network can be reused for solving different planning problems through activating different sets of context neurons which encode different goals and constraints (e.g., reaching for different goals or avoiding obstacles).

The presented theory is compatible with recent results on transient firing observed in behaving animals in phases of rest^10^,^11^,^12^,^13^. In particular, the model reproduces characteristic spatiotemporal spiking activity that preplays movement paths to remembered home locations^10^ and provides an alternative to attractor networks^14^, which are typically used to explain movement planning in maze navigation tasks^15^,^16^,^17^,^18^. In attractor networks only the end point can be adapted. For non-straight line paths, for example to escape a labyrinth, complex attractor sequence models were proposed^19^. The presented neural model allows to learn such non-straight line paths from single scalar rewards that encode in addition to desired goals and obstacles knowledge about rewarding routes. The ability to modulate the shape of a movement path is a testable prediction for future neuroscience studies.

Another contribution of the presented theory is that it provides the basis for neuromorphic hardware implementations of control and planning strategies in mobile robots. Similar architectures building also on winner-take-all circuits and spike-timing dependent plasticity (STDP) rules were already proposed as computational models for such brain-like chips^20^,^21^,^22^. For planning, neuromorphic hardware implementations promise to process large input streams from visual and tactile sensors through parallel computing^23^, go round strategies may be initiate in real-time to avoid dangerous situations in robot co-worker scenarios and event based neural network implementations are energy efficient alternatives to classical von Neumann architectures^22^. In first experiments, we demonstrate that spiking neural networks can be trained to simultaneously represent multiple obstacle avoiding strategies in a real robot arm reaching task.

## Methods

We first formulate finite horizon probabilistic planning tasks with terminal rewards only. Later we will generalize to infinite horizons where rewards can be received at any point in time. Let 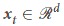 denote the *d* dimensional continuous state of a behaving agent at time *t*. The goal of the agent is to find the sequence of states 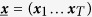 that maximizes the total received reward (i.e., the return) 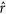 at the end of the trial.

Such planning tasks can be modeled as inference problems^1^,^2^ where the joint distribution over state sequences and returns is given by 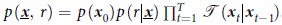. The distribution *p*(***x***_0_) encodes an initial prior over states, 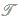 corresponds to the state transition model, and the distribution 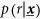 determines the probability of receiving the return *r* given the trajectory of states 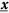. As in related work^1^,^2^,^24^ we assume that 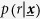 can be factorized as 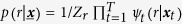. In this formulation, *r* denotes a binary random variable, where without loss of generality, the probability of observing such a binary return event is a modulo rescaling of the original reward maximization problem^1^.

An agent can use such an internal model of the environment to plan a sequence of movements by solving the inference problem


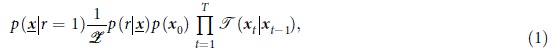


where 
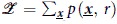
 is a term that guarantees that equation (1) is normalized. In our formulation of the planning problem, the actions in the probabilistic model are integrated out. It is assumed that the actions can be subsequently inferred from the posterior over state sequences.

The unconstrained process for planning models a freely moving agent by


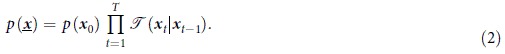


Sampling from this probability distribution can be implemented by a recurrent network of spiking neurons (e.g., using ideas from^7^,^8^,^9^). However, it is not straightforward for a recurrent network to solve the inference problem in equation (1), which requires to integrate future returns backward in time. Only local temporal information is available when sampling from the network. Such temporal models are different to model-based Markov decision process methods encoding global value or Q functions^25^.

We propose here a solution to this problem that relies on replacing 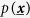 with a model distribution 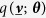, where sampling from 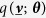 is implemented with an extended neural network architecture and 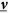 is the neural approximation of 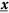. The parameters ***θ*** are learned such that the Kullback-Leibler divergence between the true posterior for planning in equation (1) and a model distribution 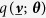 converges to zero.

### Planning with recurrent neural networks

**Figure 1:**
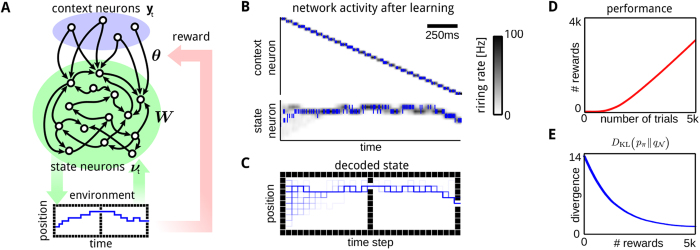
Illustration of the model for finite horizon planning. (**A**) The neural network architecture considered here for solving the probabilistic planning problem. A recurrent layer of state neurons (green) that control the behavior of the agent receive feedforward input from context neurons (blue), the activity of which determine the desired goal. (**B,C**) A simple planning problem that requires to pass through a passage at two specific points in time. Superposition of network activity averaged over 100 trial runs (**B**) and decoded network states (**C**) are shown. Blue dots in (**B**) show 1 example spike train. **(D**) The accumulated number of rewards for the spiking network. (**E**) The Kullback-Leibler divergence between the learned distribution and the true posterior. (**C**,**D**) show averages over 100 trial runs.

We propose a recurrent spiking neural network to implement planning. Our network consists of two populations of neurons, which we denote by *Y* and *V* (see Fig. 1A). *V* is a layer of *K state neurons* that control the state (e.g., the agent's spatial location) of a freely moving agent. These neurons receive lateral connections from neighboring state neurons and from all *N* neurons in a population of *context neurons Y* with weights *w*_*ki*_ and *θ*_*kj*_. The context neurons produce spatiotemporal spike patterns that represent high-level goals and context information (e.g., the target state that should be reached after *T* time steps). We show that probabilistic planning problems defined in equation (1) can be implemented in the network by training the synapses *θ*_*kj*_.

We denote the activity of the state neurons at time *t* by 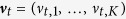, where *ν*_*t,k*_ = 1 if neuron *k* spiked at time *t* and *ν*_*t,k*_ = 0 else. Discrete random variables ***x***_*t*_ can be encoded as a multinomial distribution, where one neuron maps to one state instance. For continuous variables a simple encoding scheme is used, i.e., 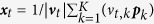, where 
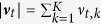
 and ***p***_*k*_ is the preferred position of state neuron *k*.

Analogously, we define the spiking activity of the context neurons at time *t* by a binary vector 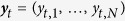. Using these definitions of ***v***_*t*_ and ***y***_*t*_, we define the membrane potential *u*_*t,k*_ and firing probability *ρ*_*t,k*_ of state neuron *k* at time *t* by


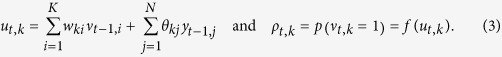


The last term *f*(*u*_*t,k*_) denotes the activation function, where we only require that it is differentiable. The probability that the network generates a spike sequences 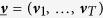 of length *T* starting from a given initial state ***v***_0_ is thus


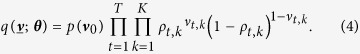


We assume that the transition model (encoded in the synaptic weights *w*_*ki*_) is known or was acquired in a pre-learning phase, e.g., using contrastive divergence learning^26^. Using this assumption, we define the goal of probabilistic planning as minimizing the Kullback-Leibler divergence between the true posterior for planning in equation (1) and the model distribution


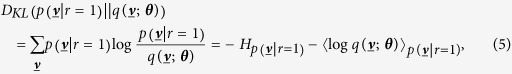


where 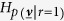 denotes the entropy of the true data distribution. Thus, solving the inference problem in equation (1) is equal to minimizing the Kullback-Leibler divergence in equation (5).

Typically, 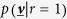 is unknown and we cannot draw samples from it. However, we can draw samples from the model distribution 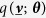 and update the parameters such that the probability of receiving a reward event is maximized


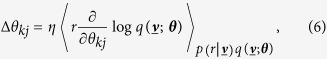


where *η* denotes a small learning rate. Note that this general update rule is the result of a standard maximum likelihood formulation where we exploited that 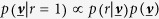 using equation (1) and equation (2). The update is an instance of the Expectation-Maximization (EM) algorithm^27^, where evaluating the expectation with respect to 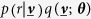 corresponds to the E-step and the parameter update realizes the M-step. The update is also related to policy gradient methods^28^,^29^,^30^ with the difference that we interpret the parameters ***θ*** as having the role of linear controls^31^.

To derive the update rule for the proposed neural network architecture, the network dynamics in equation (4) is used in equation (6). For a detailed derivation we refer to the supplement. The spiking network update rule reads


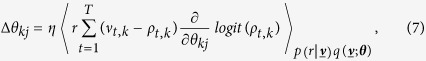


where 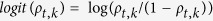 are the log-odds of neuron *k* firing at time *t*. Equation (7) is the general learning rule for arbitrary differentiable activation functions 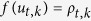. It adapts the weights *θ*_*kj*_ to maximize the return. For many relevant activation functions (e.g., exponential or sigmoid functions), equation (7) turns into a simple reward-modulated Hebbian-type update rule.

We will compare *online* and *offline* updates of equation (7). In its stochastic *online* variant, the E-step is approximated by sampling a finite set of *L* samples to estimate the expectation^32^, or in the simplest case after a single sample (*L* = 1) as done in our experiments. We refer to this as the online approximation of equation (7). With *offline* updates, implemented as batch learning, the KL divergence 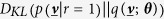 between the true posterior for planning in equation (1) and the model distribution converges to zero for *L* → ∞ (assuming an exact encoding of the state and the transition model). This KL divergence establishes the relation between the inference problem for planning in equation (1) and the introduced problem of finding the network parameters that maximize the expected return.

### A finite horizon planning task

To evaluate the spiking neural network model we consider a simple one dimensional planning problem, where the agent moves on a linear track and the activity of the state neurons population *V* directly determines its position. *K* = 9 state neurons encode nine discrete locations. A final reward is only received if the agent passes through two obstacles, one at time *T*/2 and one at time *T* (see Fig. 1C). Furthermore the agent is constrained not to jump to distant states within one time step. We model this constraint by the state transition model, i.e., 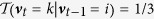, if 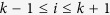 and (close to) zero otherwise (see the supplement for further details).

Due to the limitation on the state transitions, this problem requires planning ahead in order to avoid the obstacles successfully, i.e., to start moving to the passage before the obstacle actually appears. We show that the optimal planning policy can be learned using the reward modulated update rule in equation (7) in a network where the state neurons follow (soft) winner-take-all (WTA) dynamics. The probability *ρ*_*t,k*_ of neuron *k* to spike at time *t* is given by 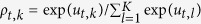. Thus, in each time step exactly one state neuron is active and encodes the current position of the agent.

The precise timing required to solve this task can be learned if the context neurons provide sufficient temporal structure. We study here the case of only one context neuron being active for one time-step, i.e., 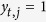 for *j* = *t* and 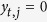 else. The weights *θ*_*kj*_ were adapted according to the online approximation of equation (7). Prior to learning, the agent performs a random walk according to the state transition model encoded in the weights *w*_*kj*_, performing successful trials only occasionally. As learning proceeds, the activity of the context neurons shapes the behavior of the agent leading to nearly optimal performance. Figure 1D shows the accumulated reward throughout learning. After 5000 training iterations, the network generates rewarded trajectories in 97.80 ± 4.64% of the trials. We also evaluated a more detailed spiking version of the network model which produced similar results (success rate: 87.40 ± 15.08%, see Fig. 1B,C in the supplement).

In addition to the online learning rule, we also evaluated the offline update rule. The network draws samples from a fixed distribution 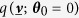 simulating random walks without any input (the initial state distribution *p*(***v***_0_) was uniform). Offline updates are applied to the parameters ***θ*** and Kullback-Leibler divergence converges towards zero with an increasing number of updates as shown in Fig. 1E.

### Extension to the infinite horizon problem

Previously, we demonstrated how our network can model finite horizon planning tasks with terminal rewards (i.e., returns). Here we generalize to infinite horizon planning problems where rewards can be received at any point in time. The goal of the planning problem is to optimize the parameters ***θ*** in the neural network so that it generates infinite trajectories that maximize the expected total discounted reward 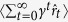, where *γ* is the discount factor. We can reformulate this planning problem as probabilistic inference in an infinite mixture of Markov chains of finite lengths *T*^1^. The corresponding mixture distribution of trajectories is given by


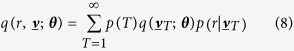


where 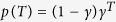 is the prior distribution over trajectory lengths, and 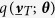 is the distribution over spike trains of length *T* according to the network dynamics in equation (4). The probability of getting a reward *r* at the end of the trajectory is given by 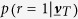. Intuitively, in infinite horizon planning tasks the agent seeks a solution that balances getting to the goal as fast as possible (imposed by the prior) against the cost of large state jumps (imposed by the state transition model).

For the infinite horizon model we consider network dynamics where each state neuron has a sigmoid activation function, i.e., 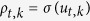 with 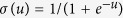. Using this activation function we find that for learning the infinite planning task the parameters *θ*_*kj*_ should undergo a change in each time step *t* where the reward is present, according to


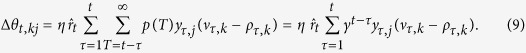


This synaptic weight update can be realized using an eligibility trace^33^ *e*_*t,kj*_ associated to each synapse with dynamics


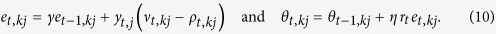


The eligibility trace is updated in each time step, whereas the weight updates are only applied for 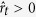. More details on the learning rule can be found in the supplement.

Note that the precise timing of attracting or repelling states cannot be modeled through explicit context neurons per time step as in the finite horizon model (since 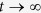). Therefore, we consider stationary activity patterns of context neurons. This assumption implies that after convergence of the parameter updates an attractor cannot be visited twice.

### An infinite horizon planning task

To test the infinite horizon model we consider a planning task, where the goal of the agent is to navigate from a given initial state to a target state in a grid maze with obstacles (dimensions [15 × 20]). The network has 300 state neurons, one for each grid cell. The agent can perform only one-step moves, to the left, to the right, up or down, with equally probable transitions in each direction, and receives a reward 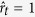 only at the target state. The sampling process is either terminated if the target state is reached, or if the time step exceeds the maximum number of allowed steps (*T* = 300). The discount factor was *γ* = 0.98.

**Figure 2:**
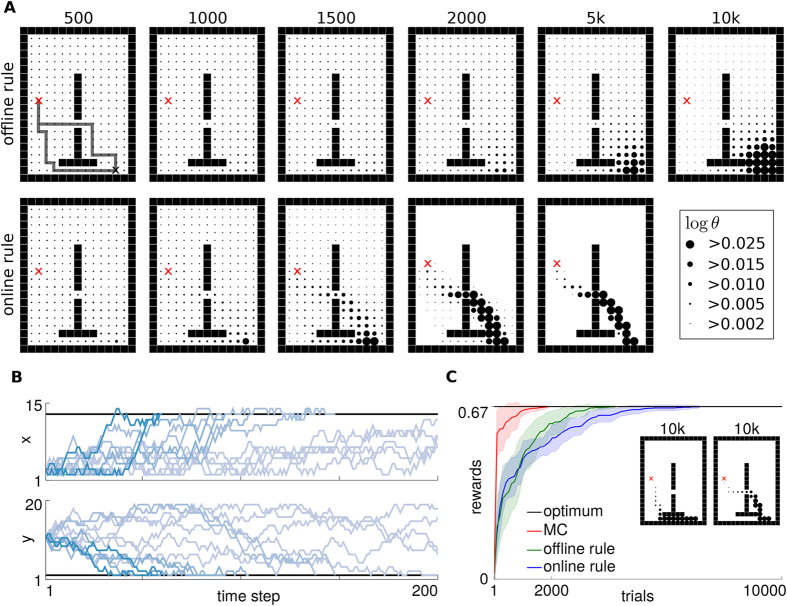
Illustration of the model for infinite horizon planning. (**A**) The agent has to move from the red cross the black cross. The radii of the dots are proportional to the log of the ***θ*** parameters. The results for the offline and the online learning rules are shown in the two rows, respectively. (**B**) Illustration of 12 sampled trajectories after 10000 trials of offline learning. (**C**) The mean of the received rewards over 20 experiments. We compare to Monte-Carlo policy evaluation (MC).

With the offline learning rule the learned parameters ***θ*** setup a gradient towards the target state, which covers *multiple* solution trajectories that lead to high total received rewards (***θ***_0_ is chosen such that the agent starts at the initial state). This gradient is indicated by the radii of the dots in the first row in Fig. 2A. Figure 2B illustrates 12 example trajectories with weights obtained after 10000 trials of learning. Out of the shown 12 trajectories 9 reached the target state that is denoted by the black horizontal lines.

With the online learning rule, the learned parameters ***θ*** specialize on one locally optimal path through the maze, which is illustrated in the second row of Fig. 2A. In the evaluated example, there are two locally optimal trajectories that are also the global optima. They are shown in the inset in Fig. 2C. For both, the offline and the online updates the average received reward converges to the maximum value (see Fig. 2C), where we compare to Monte-Carlo policy evaluation (MC)^25^.

## Results

### A computational model for hippocampal sweeps

**Figure 3:**
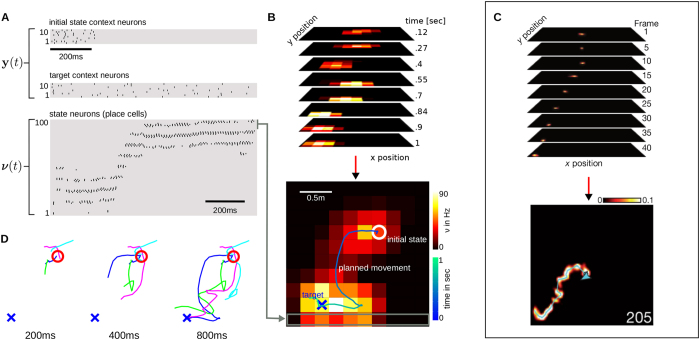
Planning and modeling transient firing in place cells with simulation results in (**A**,**B**,**D**). (**C**) Biological data showing the position posterior probabilities in selected frames (the event duration was 205 ms). Reprinted by permission from Macmillan Publishers Ltd: *Nature* 497, 74–79, ©2013. (**A**) The first ten context neurons are only activated for the first 200 ms to initialize the network at the desired initial state. The ten context neurons in the 2nd row in (**A**) implement a gradient towards the desired target state. The transient neural activity of 100 place cells is shown in the 3rd row in (**A**). The reconstructed movement trajectory is denoted by the line in (**B**). (**D**) The result of four planning processes for an increasing planning horizon.

We show that the neural network reproduces the transient firing recorded in place cells in rats during planning phases. We compare our model predictions to recent results in^10^, where the authors analyzed the neural activity of 250 simultaneously recorded hippocampal neurons during phases of mental planning (using a 40-tetrode microdrive). In the experiments the animals received a reward, in an alternating manner, either at a known home location or at some (unknown) random location (one out of 36 locations arranged in a grid in a 2 × 2 m maze). Here, we model only events that were observed while the animal was *resting* at some known current location and plans a route to the memorized home location (mental planning). An example event of a rat is illustrated in Fig. 3C, which illustrates the transient in the reconstructed position posterior probabilities. The resulting movement plan, i.e., the decoded and summed place cells’ activity across time is shown in the lower panel in Fig. 3C.

In our network, the current location and the target location of the rat are modeled by *N* = 20 context neurons. The activity of which is denoted by **y**(*t*) and shown in Fig. 3A. The first ten context neurons encode the current location through a transient pattern. The remaining ten context neurons represent the desired target location. These neurons fire with a stationary Poisson process throughout the experiment. In this experiment we did not learn the weights of the context neurons. The weights were set proportional to the Euclidean distance of the preferred position of place cells to the initial state or the home location.

The network activity of 100 place cells (the preferred positions of which are aligned with a 10-by-10 grid) is solely driven by the context neurons and the recurrent weights. The recurrent synapses implement a Gaussian state transition model which prevents that the network directly draws samples close to the target state. In contrast to the ideal model used in the previous experiments multiple place cells can be active simultaneously, i.e., the recurrent weights encode a soft WTA circuit as in^34^.

#### Encoding continuous state variables

A simple encoding scheme is used to reconstruct the two dimensional state of the simulated rat ***x***(*t*) from the place cells' activity, i.e., 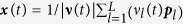, where 
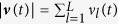
 and ***p***_*l*_ is the preferred position of place cell *l*.

The generated transient activity of the place cells realize a path between the initial and the home location as shown in the 3rd row in Fig. 3A. The reconstructed movement plan is denoted by the line in Fig. 3B, where the integrated activity over the whole movement duration of each place cell is encoded by the color of the corresponding grid position. The simulated sequential firing shows a coherence to the transient firing in place cells in rats that is illustrated in Fig. 3C (see also the online material in a work of B. Pfeiffer and D. Foster^10^). A notable difference from the biological data is the resolution of the simulated activity in Fig. 3B. To visualize the corresponding spike events only 100 place cells were modeled. For a higher density of 900 uniformly distributed place cells we refer to additional results provided in the supplement.

### Task adaptation through context neurons in a real robot

Typical robot planning tasks have to consider a large number of constraints that dynamically change and planning algorithms have to adapt to new solutions online. Here, we show that by activating context neurons multiple constraints can be modeled and used for task adaptation. As test platform we used a KUKA lightweight arm controlled in a two dimensional Cartesian space. The network generated movement plans in 2*D* and the complexity of the control problem itself is absorbed by a built-in Cartesian tracking controller. The two coordinates modeled, *x* and *y* span the transverse plane. The trajectory was executed using inverse kinematics to obtain a reference joint trajectory and inverse dynamics control to execute it.

**Figure 4:**
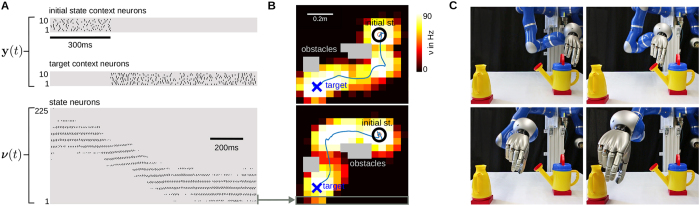
Planning with multiple constraints on a real robot. (**A**) Generated spike train (top: context neurons, bottom: state neurons) after contrastive divergence learning of the transition model. (**B**) Two sampled movement plans solving the obstacle avoidance task. (**C**) Snapshots of the executed movement on the real robot.

We used *K* = 225 state neurons that receive excitatory input from context neurons modeling the initial state and the target state (as in the previous experiment), see Fig. 4A. Strong inhibitory input is used to model obstacles (the gray areas in Fig. 4B). The transition model was learned from demonstrated state transitions (through kinesthetic teaching) in contrastive divergence learning^26^ (see the supplement for details).

After learning, the network generates goal-directed movement plans that can be used for obstacle avoidance. Sampling a movement plan of a duration of 1.3 seconds took on average 635 ± 8 milliseconds on a standard computer (the symbol ± denotes the standard deviation computed from 1000 trajectories). The minimum distance to the target was 2.71 ± 2.24 cm in an operation area of 70 × 70 cm.

An interesting property of the spiking model is that multiple solutions can be encoded in the same model. For the considered obstacle avoidance task, two solutions are shown in Fig. 4B and snapshots of the second solution are depicted in Fig. 4C.

## Discussion

The brain efficiently processes and predicts sequential activity patterns in the context of planning tasks^10^,^11^,^12^,^13^. Understanding these processes and how information is possibly encoded in stochastic neurons are major goals of theoretical neuroscience. In this paper, we demonstrated how recurrent spiking neural networks can solve planning problems and provide a solid theory based on the framework of probabilistic inference. The model reproduces observed neural dynamics, predicts that contextual information is one of the key modulating factors and is a promising low-energy control strategy for mobile robot applications.

### Theoretical contributions

Spiking neural networks are a reasonable neuroscience model as verified by data^35^,^36^,^37^,^38^. Their capabilities in solving planning tasks however have been underexplored to date. It was shown that spiking networks can encode and draw samples from arbitrary complex distributions^39^,^40^, models of temporal sequences can be learned^7^,^8^ and Bayesian filtering was studied^38^,^41^,^42^,^43^.

Our model builds on these probabilistic sampling results and a solution to planning problems is suggested that approximates a stochastic process for planning through forward sampling from a parameterized model distribution. The parameters of the model distribution denote the synaptic weights of a population of afferent neurons for which local Hebbian update rules were derived from the principle of probabilistic inference. The derivations include arbitrary differentiable activation functions and postsynaptic potential shapes for the neuron model (details are provided in the supplement).

Links to expectation maximization^27^,^32^ and policy gradient methods^28^,^29^,^30^ were established, where the resulting offline learning rules are similar to Monte Carlo policy evaluation^25^ and the network parameters subject to these updates converge to the globally optimal solution for WTA network dynamics. The online learning rules resemble the online Monte Carlo policy iteration algorithm and converge to local lower bounds of the optimum.

The correctness of the neural planning method was validated in two toy tasks, a finite horizon planning task with a known optimal solution and an infinite horizon task, where the neural network achieved the same performance level as Monte-Carlo policy evaluation^25^.

### Implications for neuroscience

Previous neural models that implement path planning have focused on attractor networks or potential fields^15^,^44^,^45^,^46^,^47^ and their activity was related to hippocampal firing^17^,^18^. A deficit of these models is however that the path which was taken to reach a desired state cannot be modeled with attractors. To overcome this limitation a sequence of successive metastable states in attractor networks was proposed^19^ but it is left unclear how these networks can be trained from rewards. We followed a different approach where attractors emerge through reinforcement of rewarding trajectories. As a result, different input neurons with its synaptic weights can model different routes to multiple attractors. Thus, the proposed model extends the modulation abilities of attractor networks and can be validated, e.g., in a study on contrasting planning of safe versus straight-line paths.

We demonstrated in simulation results that the proposed recurrent neural network can reproduce the dynamically changing firing rates observed during hippocampal sweeps^10^. The input modulated activity in our network hypothesizes that a cognitive map representation (the recurrently connected state neurons) receives contextual input from other brain regions. Potential sites for these contextual inputs are projections from the entorhinal and the prefrontal cortex^48^,^49^. It is worth mentioning that the network is not limited to model hippocampal sweeps. It may be used to model frequently observed dynamically changing firing rates from other brain regions^50^,^51^,^52^,^53^.

Embedded in the framework of probabilistic inference, the proposed network can be naturally extended in multiple ways, e.g., the place cells encoding the state transition model might be learned^17^, actions might be encoded additionally^34^, forward and backward replays^11^,^12^ can be simulated, or multiple cognitive maps can be installed^18^. Furthermore, Poisson neurons were chosen for simplicity and the model generalizes to noisy integrate and fire neurons^54^.

### Implications for robotics

State-of-the-art planning algorithms in robotics generate movement plans within seconds and scale to many degrees of freedom^55^,^56^,^57^. Spiking neural networks could not compete with these methods due to the encoding of continuous variables, e.g., in our robot experiment, we used population codes^58^,^59^ to encode a two-dimensional continuous state variable and more than a few hundred neurons could not be simulated without risking to run out of memory in a standard computer. A potential solution that is currently under investigation are factorized population codes which scale but can not capture correlations (which are needed to avoid obstacles). Another promising alternative are neuromorphic chips which were already used to learn 62-dimensional joint angle sequences of human jump motions in recurrent networks^60^. In addition, related spiking network models were proposed which also build on winner-take-all circuits and local plasticity rules^20^,^21^,^22^. Therefore, it is reasonable to assume that the presented theory provides the basis for future neural controller implementations in neuromorphic hardware for robot control.

In contrast to previous work on spiking neurons in a reinforcement learning framework^34^, we followed here a model-based approach where the recurrent dynamics of the network can be reused to learn multiple related tasks with different sets of weights from the context neurons (e.g., representing different goal positions or obstacles). The state transition model does not need to be re-learned when switching between environments.

Furthermore, our model has the advantage that multi-modal solutions to planning tasks can be learned. This feature was exploited in an obstacle avoidance task in a real robot, where the network randomly sampled one out of two paths. This ability to encode non-linear mappings is in particular beneficial for learning forward and inverse kinematic models in robotics.

## Additional Information

**How to cite this article**: Rueckert, E. *et al*. Recurrent Spiking Networks Solve Planning Tasks. *Sci. Rep.* **6**, 21142; doi: 10.1038/srep21142 (2016).

## Supplementary Material

Supplementary Information
