# Supplementary material for: Recurrent Spiking Networks Solve Planning Tasks
Source: Sci Rep. 2016 Feb 18;6:21142. doi: 10.1038/srep21142 (PMC4758071; doi:10.1038/srep21142)
Supplement: Supplementary Information [file srep21142-s1.pdf]

Supplement to Recurrent Spiking Networks Solve Planning Tasks

Elmar Rueckert[1,*], David Kappel[2], Daniel Tanneberg[1], Dejan Pecevski[2], and Jan Peters[1,3]

[1]Intelligent Autonomous Systems Lab, Technische Universität Darmstadt, 64289, Germany
[2]Institute for Theoretical Computer Science, Technische Universität Graz, 8020, Austria
[3]Robot Learning Group, Max-Planck Institute for Intelligent Systems, Tuebingen, 72076, Germany
[*]rueckert@ias.tu-darmstadt.de

ABSTRACT

This supplement provides additional material to the model derivations, to the maze navigation task and the robot control experiment.

Details to *Planning with recurrent neural networks*

Here we provide a detailed derivation of the general learning rule in equation (7) in the main text. The derivations include arbitrary differentiable activation functions and postsynaptic potential (PSP) shapes for the neuron model. All applications of the finite horizon model in the main paper can therefore be seen as special cases of the theoretical framework presented here.

Therefore, we consider a neuron model with arbitrary synaptic dynamics that inject at time t postsynaptic currents $\tilde{v}_i(t)$ and $\tilde{y}_j(t)$ in response to spike trains $\underline{\boldsymbol{v}}$ and $\underline{\boldsymbol{y}}$ into neuron k. Thus, $\tilde{v}_i(t)$ and $\tilde{y}_j(t)$ amount for the effect of PSPs on the lateral and feedforward synapses, respectively. The membrane potential is then given by

$$u_k(t) = \sum_{i=1}^{K} w_{ki}\,\tilde{v}_i(t) + \sum_{j=1}^{N} \theta_{kj}\,\tilde{y}_j(t)\ . \tag{S1}$$

Equation (S1) is a simple stochastic version of the spike response model.[1] Equation (3) of the main text is a special case of this form with a resetting rectangular PSP kernel which is of one time step length and thus $\tilde{v}_i(t) = v_i(t)$ and $\tilde{y}_j(t) = y_j(t)$.

Solving the inference problem specified in equation (1) in the main text is equal to minimizing the Kullback-Leibler divergence defined in equation (5) in the main text. This requires to compute the derivative of the log-probability over spike sequences $\frac{\partial}{\partial \theta_{kj}} \log q(\underline{\boldsymbol{v}}; \boldsymbol{\theta})$. Inserting (4) from the main text allows to rewrite this derivative

$$\begin{aligned}
\frac{\partial}{\partial \theta_{kj}} \log q(\underline{\boldsymbol{v}}; \boldsymbol{\theta}) &= \frac{\partial}{\partial \theta_{kj}} \sum_{t=1}^{T} \big(v_{t,k} \log \rho_{t,k} + (1 - v_{t,k}) \log(1-\rho_{t,k})\big) && \text{(S2)}\\
&= \frac{\partial}{\partial \theta_{kj}} \sum_{t=1}^{T} \left(v_{t,k} \log\left(\frac{\rho_{t,k}}{1-\rho_{t,k}}\right) + \log(1-\rho_{t,k})\right) && \text{(S3)}\\
&= \sum_{t=1}^{T} \big(v_{t,k} - \rho_{t,k}\big) \frac{\partial}{\partial \theta_{kj}} \log\left(\frac{\rho_{t,k}}{1-\rho_{t,k}}\right) && \text{(S4)}\\
&= \sum_{t=1}^{T} \big(v_{t,k} - \rho_{t,k}\big) \frac{\partial}{\partial \theta_{kj}} \operatorname{logit}(\rho_{t,k})\ , && \text{(S5)}
\end{aligned}$$

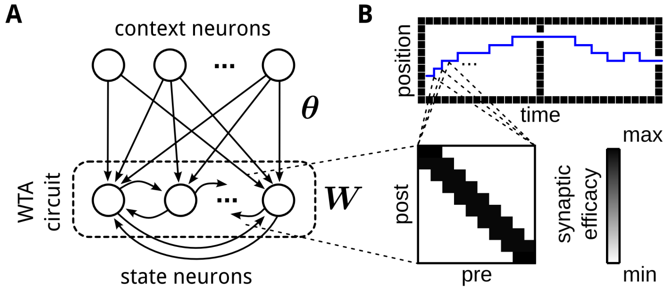

Figure S1. Illustration of the simple probabilistic planning model. **A:** The neural network architecture considered here for solving the probabilistic planning problem. A layer of state neurons that control the behavior of the agent receive feedforward input from context neurons, the activity of which determine the desired goal. **B:** A simple planning problem that requires to pass through a passage at two specific points in time. One successful movement trajectory is shown (blue). The inset shows the lateral synaptic weights between the state neuron that encode the transition model for this task.

where $\text{logit}(\rho) = \log\left(\frac{\rho}{1-\rho}\right)$ is the log-odds function. In (S4) we used $\frac{\partial}{\partial\theta}\log(1-\rho) = \frac{-1}{1-\rho}\frac{\partial}{\partial\theta}\rho = \left(1-\frac{\rho}{1-\rho}\right)\frac{\partial}{\partial\theta}\rho = \rho\frac{\partial}{\partial\theta}\left(\log(\rho)-\log(1-\rho)\right)$. The general update rule in equation (7) in the main text follows then directly from this last result.

Detail to *A finite horizon planning task*

Here, we provide additional details to the network model that was used to solve the finite horizon planning model in Fig. S1A. In this architecture the activity of the K state neurons was constrained to winner-take-all (WTA) dynamics, which assures that exactly one neuron is active in each time step, i.e., $\sum_k v_{t,k} = 1, \forall t$. Therefore the spiking probability $\rho_{t,k}$ of neuron k to spike at time t is given by

$$\rho_{t,k} \;=\; p\left(v_{t,k}=1 \,\middle|\, \boldsymbol{v}_{t-1}, \boldsymbol{y}_t; \theta\right) \;=\; \frac{\exp\left(u_{t,k}\right)}{\sum_{l=1}^{K}\exp\left(u_{t,l}\right)}\;. \tag{S6}$$

The WTA network gives rise to a very simple planning model. The state neurons directly encode the discrete state of the environment, where $v_{t,k}=1$ denotes that the environment is in state k at time t. Furthermore, the lateral synaptic weights directly encode the transition operator $\mathcal{T}$ of this planning model. To see this, we note that the dynamics realize a distribution over network state trajectories $\boldsymbol{v}_{1:T}$, which can be written in the form

$$q\left(\boldsymbol{v}_{1:T} \mid \boldsymbol{\theta}\right) \;=\; p(\boldsymbol{v}_0)\prod_{t=1}^{T}\prod_{k=1}^{K}\rho_{t,k}{}^{v_{t,k}}\left(1-\rho_{t,k}\right)^{1-v_{t,k}} \tag{S7}$$

$$\;=\; p(\boldsymbol{v}_0)\prod_{t=1}^{T}\mathcal{T}\left(\boldsymbol{v}_t \mid \boldsymbol{v}_{t-1}\right)\phi_t\left(\boldsymbol{v}_t\,;\boldsymbol{\theta}\right)\;, \tag{S8}$$

where by inserting equation (3) of the main text we identify

$$\mathscr{T}\left(\boldsymbol{v}_t \mid \boldsymbol{v}_{t-1}\right) = \exp\left(\sum_{i=1}^{K} w_{ki}\, v_{t-1,i}\, v_{t,k}\right),$$

$$\phi_t\left(\boldsymbol{v}_t\,;\boldsymbol{\theta}\right) = \frac{\exp\left(\sum_{j=1}^{N} \theta_{kj}\, y_{t-1,j}\, v_{t,k}\right)}{\sum_{l=1}^{K} \exp\left(u_{t,l}\right)}\,.$$

Thus the first term of (S8) determines the transition operator $\mathscr{T}$ implemented through the lateral weights w_{ki}, and the second term realizes the function ϕ_t parametrized by the feedforward weights θ_{kj}. From this we can see that the weights w_{ki} encode the log-probabilities of making a transition from state i to state k, i.e., $w_{ki} \propto \log \mathscr{T}\left(v_{t,k}=1 \,\middle|\, v_{t-1,i}=1\right)$ of the planning model.

To derive the update rules for the feedforward weights θ_{kj} projecting onto the WTA network, we plug in the activation function (S6) into the general form in equation (S5). Using this we find that the weights θ_{kj} become optimal, subject to the learning rule

$$\Delta\theta_{kj} = \eta \left\langle r \sum_{t=1}^{T} y_{t-1,j}\left(v_{t,k} - \rho_{t,k}\right)\right\rangle_{p(r\,|\,\boldsymbol{v}_{1:T})q(\boldsymbol{v}_{1:T}\,;\boldsymbol{\theta})}\,. \qquad \text{(S9)}$$

The updates converge to a lower bound of the KL-divergence, see (5) in the main text. If the activity of the context neurons has the following temporal structure this bound becomes tight and the KL-divergence vanishes: We assume that in each time step exactly one context neuron is active, i.e., $N=T$ and $y_{t,j}=1$ if $t=j$. This allows the network to precisely learn the temporal structure of the planning task. To see this we plug in the assumed dynamics of the context neurons and (S6) into equation (S9) and set it to zero, which yields the fixed point θ^*_{kj} of parameter θ_{kj} given by $\theta^*_{kj} \propto \log p(v_{k,j}=1|r=1)$. The parameters θ_{kj} converge (up to a normalizing constant) to the probability that neuron k should spike at time $t=j$, given that the reward is acquired at the end of the trial.

We evaluated the model in the simple navigation task illustrated in Fig. S1. FigureS1A illustrates the WTA network architecture. As described above, the state neurons encode the current state of the agent, which can be at any position on a linear track with $K=9$ different locations. Each trial has a duration of $T=30$ time steps. A reward is delivered to the agent if it avoids an obstacle at time $T/2$ and at time T. The planning problem and the synaptic weights w_{ki} that implement the state transition probabilities are illustrated in Fig. S1B. The agent is not allowed to jump to arbitrary positions but only to neighboring states.

In the first implementation of this task we used a time-binned binary version the network that did not use the PSP kernels, i.e., $\tilde{v}_{t,k}=v_{t,k}$ and $\tilde{y}_{t,j}=v_{t,j}$. Furthermore one neuron of the network was forced to fire in each time step according to (S6). This neuron model allows to directly implement the simple planning problem and we found that we could achieve almost perfect performance with this network (reward rate averaged over 100 trial runs was $97.80 \pm 4.64\%$.

We compared this neuron model with a more realistic spiking model that lets each state neuron fire with a time-dependent Poisson firing rate of $\rho_0 \cdot \rho_{t,k}$. The constant $\hat{v}=100$ Hz is the maximum firing rate. Furthermore we used a resetting rectangular PSP kernels with 50 ms length and thus $\tilde{v}_{t,i}$ and $\tilde{y}_j(t)$ are one if a presynaptic spike occurred within the last 50 ms and zero

else. Here each trial had a duration of 1.5 seconds of equivalent biological time. This time was divided into 30 time bins of equal length of 50 ms. The activity of the 30 context neurons was determined by a firing probability that was set to 100 Hz for the duration of one bin. The decoded discrete state in the environment was assigned to the index of the state neuron with the highest firing rate in each time bin. Figure 1B-D of the main text was produced with this model. With this more detailed neural network model we achieved a reward rate of $87.40 \pm 15.08\%$ for the simple linear track task.

Details to *Extension to infinite horizon problems*

Here we provide additional details to the infinite horizon planning model. In the infinite horizon case, we assume that the length of a trial T is unknown. Therefore the agent may receive rewards at any time t during continuous exploration of the state space. The goal of planning is then to optimize the parameters $\boldsymbol{\theta}$ in the neural network so that it generates infinite length trajectories that maximize the expected total discounted reward $\langle \sum_{t=0}^{\infty} \gamma^t \hat{r}_t \rangle$, where r_t is a scalar reward received at time t and $\gamma \in [0,1]$ is a discount factor.

Also infinite horizon planning can be formalized as probabilistic inference problem as shown in.[2] The underlying generative model is an infinite mixture of Markov chains of finite lengths T. In our spiking neural network implementation, a spike train of length T, denoted here by $\underline{\boldsymbol{v}}_T$ is generated in response to the injected context neuron activity. The corresponding joint distribution of spike train $\underline{\boldsymbol{v}}_T$ and acquired rewards r is then given by

$$q(r, \underline{\boldsymbol{v}}_T\,; \boldsymbol{\theta}) = p(T)\, q(\underline{\boldsymbol{v}}_T\,; \boldsymbol{\theta})\, p(r|\underline{\boldsymbol{v}}_T) \tag{S10}$$

where $p(T) = (1-\gamma)\gamma^T$ is the prior distribution over trajectory lengths, and $q(\underline{\boldsymbol{v}}_T\,; \boldsymbol{\theta})$ is the distribution over spike trains of length T according to the network dynamics, equal to (S7). The probability of getting a binary reward r at the end of the trajectory of length T is equal to $p(r=1|\underline{\boldsymbol{v}}_T)$.

Using this the goal of maximizing the total discounted reward is equivalent to maximizing the likelihood of the reward, i.e., $p(r=1 \mid \boldsymbol{\theta})$.[3] Using equation (S10) we can write

$$q(r; \boldsymbol{\theta}) \;=\; \langle q(r, \underline{\boldsymbol{v}}_T\,; \boldsymbol{\theta}) \rangle \;, \tag{S11}$$

where the expectation is taken over all trajectories of arbitrary length. Instead of calculating exactly the expectation, in the learning rule we approximate the expectation over the posterior by drawing a single continuous sample from the network activity and update the parameters online at each time step t. This corresponds to an online Monte-Carlo EM approximation for the infinite horizon planning problem.[4]

The main idea for this derivation is that we can interpret sub-sequences from an infinite length trajectory of the network as weighted samples from (S10). For example, if during the run at time t a reward arrives ($r_t = 1$), then we can select each sequence of states ending at t as a sample weighted with the posterior probability of its length $p(T|r=1;\boldsymbol{\theta})$. Moreover, in the

stationary case (when the context neurons inject a stationary input into the state neurons) the posterior over trajectory lengths becomes independent of the parameter and we get $p(T|r=1;\boldsymbol{\theta}) = p(T)$. Therefore we can directly weight the sample spike trajectories by the prior $p(T) = (1-\gamma)\gamma^T$. This entails that for each time step t where the reward is $r_t = 1$ (in the stationary case, where t becomes large), the parameter θ_{kj} should undergo a change equal to

$$\Delta\theta_{t,kj} = \eta\, r_t \sum_{\tau=1}^{t} \sum_{T=t-\tau}^{t} p(T) \left(\nu_{\tau,k} - \rho_{\tau,k}\right) \frac{\partial}{\partial \theta_{kj}} \operatorname{logit}(\rho_{\tau,k}) \tag{S12}$$

$$\approx \eta\, r_t \sum_{\tau=1}^{t} \sum_{T=t-\tau}^{\infty} p(T) \left(\nu_{\tau,k} - \rho_{\tau,k}\right) \frac{\partial}{\partial \theta_{kj}} \operatorname{logit}(\rho_{\tau,k}) \tag{S13}$$

$$= \eta\, r_t \sum_{\tau=1}^{t} \gamma^{t-\tau} \left(\nu_{\tau,k} - \rho_{\tau,k}\right) \frac{\partial}{\partial \theta_{kj}} \operatorname{logit}(\rho_{t,k})\,. \tag{S14}$$

This is the general update rule for infinite horizon planning problems and equals to the total update of θ_{kj} from all considered sample trajectories up to time t.

The update can be realized by using an eligibility trace $e_{t,kj}$ associated to each synapse kj with dynamics that are updated in parallel to the synaptic weights

$$\begin{aligned} e_{t,kj} &= \gamma e_{t-1,kj} + \left(\nu_{t,k} - \rho_{t,k}\right) \frac{\partial}{\partial \theta_{kj}} \operatorname{logit}(\rho_{t,k})\,, \\ \theta_{t,kj} &= \theta_{t-1,k} + \eta\, r_t\, e_{t,kj}\,. \end{aligned} \tag{S15}$$

Equation (10) of the main text is the special case for sigmoid activation functions, i.e., $\rho_{t,k} = \sigma(u_{t,k}) = 1/(1+e^{-u_{t,k}})$, of this general update rule.

As in the finite horizon case, here we can also perform offline updates of parameters $\boldsymbol{\theta}$ according to (S15) which minimizes $D_{KL}(p(\boldsymbol{\nu}|r=1)||q(\boldsymbol{\nu};\boldsymbol{\theta}))$. With the offline learning rule the inputs install a gradient in the state space which integrates backward smoothing in the sampling process of trajectories. This has the effect that the stochastic trajectories are "attracted" by states in the state space with high probability of receiving a reward.

Details to *Modeling the transient firing in place cells*

A notable difference to the biological data is the resolution of the simulated activity. In the paper only 100 place cells were modeled to visualize the corresponding spike events. For a higher density of uniformly distributed place cells (i.e., 900 place cells are aligned with a 30-by-30 grid) the trend of the place cells' activity of three example movements is shown in Fig. S2. Here from left to right we illustrate the place cells' activity for seven points in time. In each of the three rows in Fig. S2, the simulated movements were generated with injected input from 30 context neurons encoding the initial state (which is the same in all three examples) and from injected input from 30 context neurons modeling different target locations. In summary, by activating different context neurons that encode different initial or target states, the model can flexible adapt to changes in the environment or could be used to model forward or backward replays.[5–7]

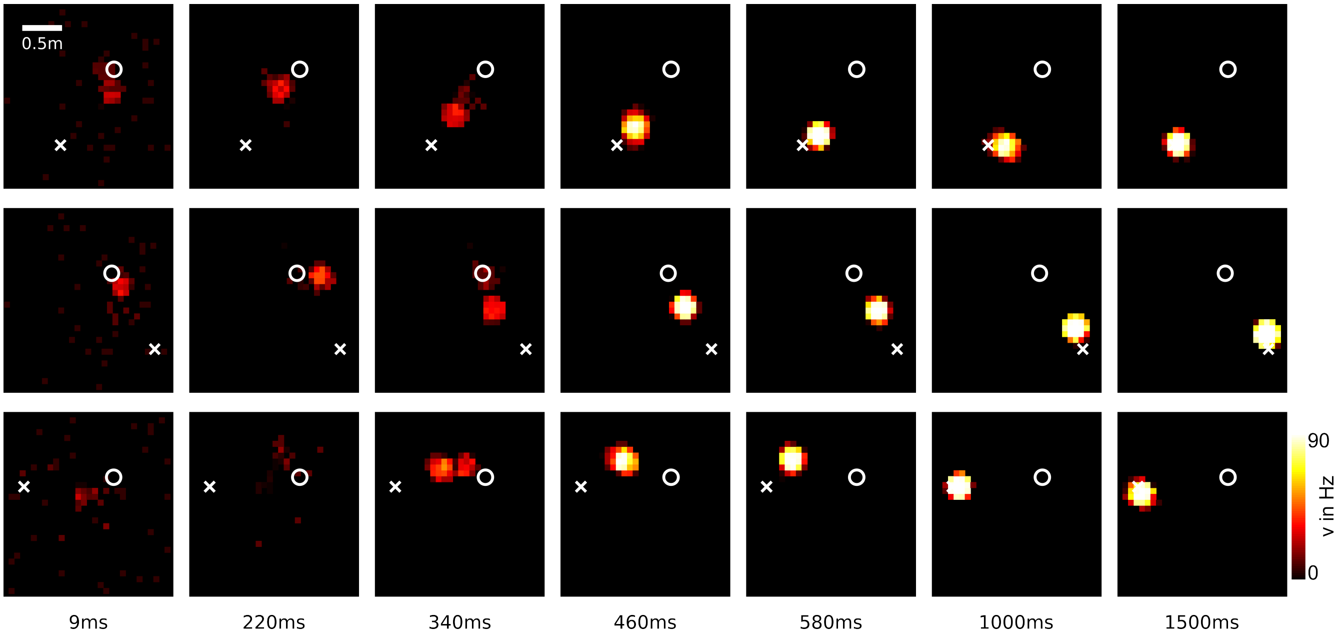

Figure S2. Illustration of three inferred movement trajectories, where the task was to move from the circle to the cross. The panels show the filtered neural activity of 900 state neurons, which represent place cells in a cognitive map (preferred positions of the 30×30 place cells are equally distributed in the grid). From left to right seven images of the neural activity are shown. The values of the firing rates of the place cells are depicted in the colorbar on the right.

Details to *Learning obstacle avoidance tasks in a real robot*

The proposed spiking neural network is a restricted Boltzmann machine for which efficient learning methods such as contrastive divergence (CD)[8] have been developed. We used CD to learn the transition model encoded in the weights w_{ki}. CD estimates a maximum likelihood gradient by drawing samples from the model distribution $q(\underline{\boldsymbol{v}};\boldsymbol{\theta})$ in equation (4) in the main text. This gradient is used to update the model parameters given by

$$\Delta w_{ki} \;=\; \alpha \left(\tilde{v}_{t-1,k}\, \tilde{v}_{t,i} \;-\; v_{t\tilde{-}1,k}\, v_{t,i}\right) \;,$$

with the learning rate $\alpha = 0.05$, demonstrations $\tilde{v}$ and model samples v. The data samples are state transitions $\tilde{v}_{t-1,k}\, \tilde{v}_{t,i}$ generated from human demonstrations in kinesthetic teach-in. Only recordings of the Cartesian x and y coordinates in a KUKA lightweight arm were used. The trajectory data was transformed into spike patterns using 15 Gaussian basis functions per dimension in an inhomogeneous Poisson process. In our experiments, we used the Gaussian bandwidth parameter $\sigma^2 = 0.03$, an additional Poisson rate offset $\lambda_{off} = -1.5$ and a refractory period of 10 ms.[9] The kinesthetic demonstrations covered the operational space of the robot (a grid of 70×70 cm). In total, 15 minutes of demonstrations sampled at 1 ms were transformed into spike patterns and used for training. The progress of the weight updates is illustrated in Fig. S3.

References

1. Gerstner, W. & Kistler, W. M. *Spiking Neuron Models* (Cambridge University Press, Cambridge, 2002).

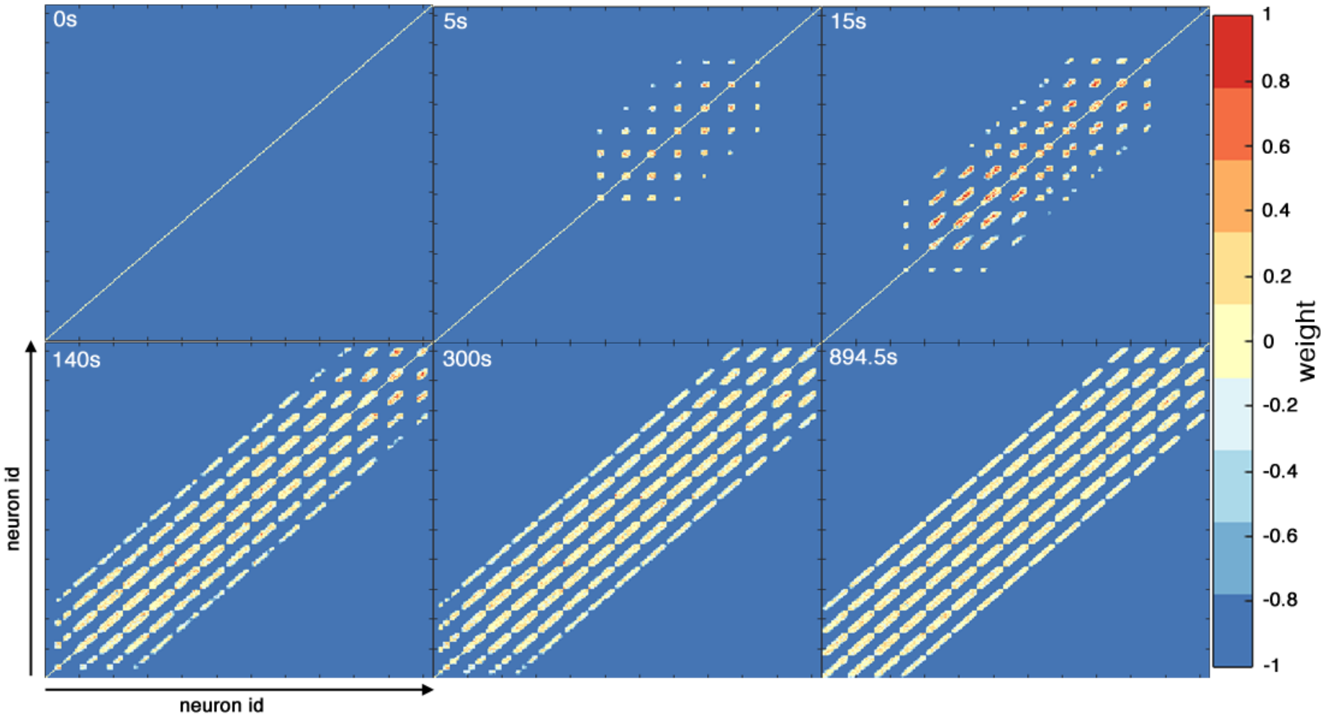

Figure S3. Illustration of learning the transition model encoded in the weights w_{ki} for $K = 225$ state neurons.

2. Toussaint, M. & Storkey, A. Probabilistic inference for solving discrete and continuous state markov decision processes. In *Proceedings of the 23rd international conference on Machine learning*, 945–952 (ACM, 2006).

3. Solway, A. & Botvinick, M. M. Goal-directed decision making as probabilistic inference: A computational framework and potential neural correlates. *Psychological Review* **119**, 120–154 (2012).

4. Wei, G. C. G. & Tanner, M. A. A monte carlo implementation of the em algorithm and the poor man's data augmentation algorithms. *Journal of the American Statistical Association* **85**, 699–704 (1990).

5. Foster, D. J. & Wilson, M. A. Reverse replay of behavioural sequences in hippocampal place cells during the awake state. *Nature* **440**, 680–683 (2006).

6. Johnson, A. & Redish, A. D. Neural ensembles in ca3 transiently encode paths forward of the animal at a decision point. *The Journal of neuroscience* **27**, 12176–12189 (2007).

7. Carr, M. F., Jadhav, S. P. & Frank, L. M. Hippocampal replay in the awake state: a potential substrate for memory consolidation and retrieval. *Nature Neuroscience* **14**, 147–153 (2011).

8. Hinton, G. Training products of experts by minimizing contrastive divergence. *Neural computation* **14**, 1771–1800 (2002).

9. Neftci, E., Das, S., Pedroni, B., Kreutz-Delgado, K. & Cauwenberghs, G. Event-driven contrastive divergence for spiking neuromorphic systems. *Frontiers in neuroscience* **7** (2013).
